# Modelling a Linker Mix‐and‐Match Approach for Controlling the Optical Excitation Gaps and Band Alignment of Zeolitic Imidazolate Frameworks

**DOI:** 10.1002/anie.201609439

**Published:** 2016-11-15

**Authors:** Ricardo Grau‐Crespo, Alex Aziz, Angus W. Collins, Rachel Crespo‐Otero, Norge C. Hernández, L. Marleny Rodriguez‐Albelo, A. Rabdel Ruiz‐Salvador, Sofia Calero, Said Hamad

**Affiliations:** ^1^ Department of ChemistryUniversity of Reading, WhiteknightsReadingRG6 6ADUK; ^2^ School of Biological and Chemical SciencesQueen Mary University of LondonMile End RoadLondonE1 4NSUK; ^3^ Department of Applied Physics I, Escuela Técnica Superior de Ingeniería Informática, Ave. Reina MercedesUniversidad de Sevilla41012SevillaSpain; ^4^ Department of Inorganic ChemistryUniversidad de GranadaAv. Fuentenueva S/N18071GranadaSpain; ^5^ Department of Physical, Chemical and Natural SystemsUniv. Pablo de OlavideCtra. de Utrera km. 141013SevilleSpain

**Keywords:** band gaps, computational chemistry, metal–organic frameworks, photocatalysis, zeolitic imidazolate frameworks

## Abstract

Tuning the electronic structure of metal–organic frameworks is the key to extending their functionality to the photocatalytic conversion of absorbed gases. Herein we discuss how the band edge positions in zeolitic imidazolate frameworks (ZIFs) can be tuned by mixing different imidazole‐based linkers within the same structure. We present the band alignment for a number of known and hypothetical Zn‐based ZIFs with respect to the vacuum level. Structures with a single type of linker exhibit relatively wide band gaps; however, by mixing linkers of a low‐lying conduction edge with linkers of a high‐lying valence edge, we can predict materials with ideal band positions for visible‐light water splitting and CO_2_ reduction photocatalysis. By introducing copper in the tetrahedral position of the mixed‐linker ZIFs, it would be possible to increase both photo‐absorption and the electron–hole recombination times.

The attractive pore structure of metal–organic frameworks (MOFs) has led to their huge success in gas adsorption and separation applications.^1^ Recently, there has been growing interest in extending the uses of MOFs to photocatalysis, as this would allow, for example, using a single material for both capture and conversion of CO_2_ in a “one‐pot” approach. The chemical diversity of MOFs can be exploited to tailor their electronic structure to the particular photocatalytic application, whereas their porous network can provide access of reactant molecules to the active sites.^2^ Some success has been achieved on MOF‐based photocatalysis in the last few years, typically involving MOFs with metal nanoparticles or complexes incorporated inside their pores, or introduced via post‐synthetic ligand modification.^3^ But there have been few reports where MOFs (without co‐catalysts) have shown intrinsic photocatalytic activity. Gascon et al. reported the use of the IRMOF family of MOFs, with band gaps tuned via linker functionalization, in photocatalytic propylene epoxidation,^4^ while Gomes‐Silva et al. have shown the potential of UiO‐66 and related MOFs for photocatalytic hydrogen generation.^5^ MOFs with intrinsic photocatalytic behavior would indeed reduce the cost and complexity of catalyst preparation, but achieving the required electronic structure and catalytic behavior without the contribution from external species is challenging.

Herein we present the findings of our computational search for MOFs with the required electronic structure to operate as single‐semiconductor, intrinsic photocatalysts in solar‐fuel synthesis reactions. The search was focused on the zeolitic imidazolate frameworks (ZIF) family, as they exhibit the required high chemical and thermal stability in aqueous solution.^6^ ZIF structures with the same topology (SOD) and tetrahedral metal (Zn), but different imidazolate‐based linkers or combination of linkers, were computationally generated. The SOD topology (Figure 1) was chosen because of its flexibility to accept a wide range of chemical compositions, in terms of both linkers and metals.6b Recently, ZIFs with SOD topology have been experimentally investigated and shown promise in the context of photocatalytic applications.^7^ Also, its primitive cell is small enough to allow an efficient screening of a range of compositions using quantum‐mechanical calculations.

**Figure 1:**
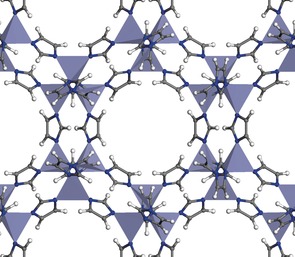
ZIF with composition ZnIm_2_ and SOD topology, viewed along the direction of the largest pore. The tetrahedra are centered on Zn atoms; N blue, C gray, H white balls.

We initially considered ZIF structures with composition ZnX_2_ and 12 different linkers (X), including the bare imidazolate (Im) linker as well as the following functionalizations: added nitro (nIm), carboxaldehyde (Ica), or methyl (mIm) side chains at ring position 2; di‐cyanide (dcnIm) or di‐chlorine (dcIm) at positions 4,5; fused benzene (bIm), pyridine (abIm, acIm) or purine (pur) 6‐membered rings; and fused thiophene (tIm) or furan (fIm) 5‐membered rings (Figure 2 a). ZIF structures with some of these linkers have been reported before, including Cu(Im)_2_, Zn(Ica)_2_, Zn(bIm)_2_, Zn(mIm)_2_ and Co(nIm)_2_ with SOD topology, as well as Zn(abIm)_2_ and Zn(pur)_2_ with LTA topology.6b To date, no ZIFs have been synthesized with tIm and fIm linkers, although these molecular units are reported in the patent literature.^8^

**Figure 2:**
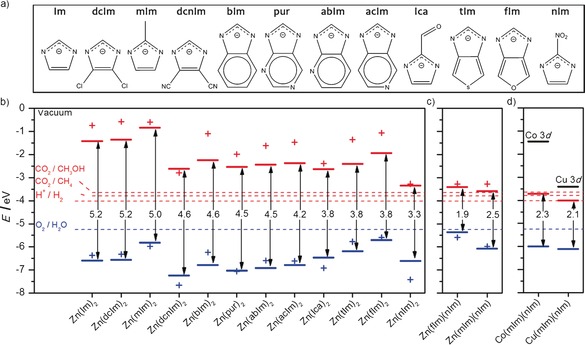
a) List of linkers (X) in the ZIF structures with composition ZnX_2_. b) Positions of the highest occupied energy levels (blue) and lowest unoccupied energy levels (red) of the ZnX_2_ crystal structures (lines), and of the isolated HX molecules (crosses). The potentials for H_2_O splitting and two different CO_2_ reduction reactions are shown as dashed lines. c) Equivalent plot for the mixed ZIF structures created by combining nIm with mIm or fIm linkers; the crosses represent the higher HOMO (blue) and the lower LUMO (red) among the two mixed linkers. d) Equivalent plot for mixed mIm/nIm ZIF structures with Co or Cu centers.

The electronic structure was determined using density functional theory (DFT) with a screened hybrid density functional,^9^ and the electronic levels were aligned with the vacuum scale.^10^ The band edges in these molecular solids, which are better referred to as the highest occupied crystal orbital (HOCO) and lowest unoccupied crystal orbital (LUCO) levels, are shown in Figure 2 b. The widest HOCO–LUCO gap is obtained in the case of the bare imidazole linker, that is, for Zn(Im)_2_ (5.2 eV), while the narrowest gap corresponds to Zn(nIm)_2_ (3.3 eV). The wide range observed illustrates the tunability of the electronic properties of ZIFs upon linker modification, which is consistent with previous theoretical and experimental findings for other MOFs families.^11^

For applications in photocatalysis, not only the magnitude of the band gap but also the absolute positions of the band edges are important. For water splitting with a single‐semiconductor photocatalyst, the band edges should straddle the redox potentials for water photolysis,^12^ that is, the HOCO should be below the energy level of the oxygen evolution reaction (OER: H_2_O↔2 H^+^_(aq)_+1/2 O_2(g)_+2 e^−^), and the LUCO should be above the energy corresponding to the hydrogen evolution reaction (HER: 2 H^+^_(aq)_+2 e^−^↔H_2(g)_). In the vacuum scale, and at pH 0, the HER level is located at −4.44 eV, and the OER level is located at −5.67 eV.^13^ At temperature *T* and pH>0, these levels are shifted up by pH×(*k*_B_ *T*×ln10), where *k*_B_ is Boltzmann's constant. In the case of CO_2_ conversion to fuels (e.g. methanol), the LUCO must be above the redox potential for the CO_2_ reduction half‐reaction.^14^ Figure 2 b shows that the HOCO and LUCO levels of the ZnX_2_ ZIF structures considered here straddle the redox levels for both water splitting and methane/methanol synthesis. However, for these photocatalysts to work efficiently under solar radiation, a narrower band gap of around 2 eV is desirable, as this would allow the absorption of the visible range solar spectrum, which carries most of the solar radiation energy. Furthermore, the band gaps of these single‐linker ZIFs correspond to intra‐linker excitations, which is not convenient for photocatalysis, because some degree of electron–hole separation is needed to prevent fast recombination of the charge carriers.^15^

We have therefore gone a step further and generated new hypothetical ZIFs by mixing different linkers within the same structure, to tailor the band edges to the required positions and to attain electron‐hole separation via inter‐linker excitations. We have chosen to mix a low‐LUCO linker (X=nIm) with high‐HOCO linkers (Y=Im, mIm, or fIm) to form ZnXY structures with potential low‐energy inter‐linker excitations. In these 50:50 mixed‐linker structures, two well‐defined ordered configurations can be formed, considering the symmetry of the lattice. For each composition, the lowest‐energy mixed configuration is significantly stable with respect to the separated pure phases (by 0.2–0.4 eV per formula unit, see Supporting Information), which indeed suggests they should be able to form experimentally. It is known that mixed‐linker ZIFs can be synthesized solvothermally, via the reaction of equimolar amounts of the two protonated linkers with the divalent metal nitrate; this has been demonstrated by Yaghi et al.,^16^ who reported the formation of structures with ordered distributions of linkers.^17^ Figure 2 c shows the HOCO and LUCO levels for the most stable mixed ZIFs with compositions Zn(fIm)(nIm) and Zn(mIm)(nIm). They perfectly straddle the targeted redox potentials and lead to gaps of 1.9 and 2.5 eV, respectively, which are very convenient for applications involving solar‐light absorption. The projected electronic density of states (DOS) in the mixed ZIFs (see Supporting Information) confirms that the LUCO is mainly contributed by the nitro group in the nIm linker, while the HOCO is contributed by the second linker.

This linker “mix‐and‐match” method can then be used more generally to target desired electronic structures of ZIFs for photocatalytic applications. The reason why the procedure works becomes apparent from examining the energies of the highest‐occupied (HOMO) and lowest‐unoccupied (LUMO) molecular orbitals of the isolated linkers. The HOMO and LUMO of the neutral molecules HX are represented as crosses in Figure 2 b (and also in Figure 2 c, but using the higher HOMO and the lower LUMO among the two mixed‐in linkers). Despite some fluctuations, the HOCO/LUCO levels in the ZIFs follow the same trend as the HOMO/LUMO levels in the corresponding gas‐phase linker molecules. As Figure 3 shows, there is an excellent correlation between the excitation gaps in the linkers and those in the corresponding ZIFs. The correlation extends to the mixed‐linker ZIFs, if we take the difference between the higher HOMO and the lower LUMO between the two linkers. It is clear from this analysis that the positions of the band edges in the crystalline material are determined, to a large extent, by the frontier orbitals of the linkers. We have compiled a larger list of modified imidazolate linkers, and calculated their HOMO and LUMO, which are provided in the Supporting Information. The motivation is that this list can serve as a reference for the electronic structures of functionalized ZIF linkers, and as a starting point for future design of mixed‐linker ZIFs with tailored bands.

**Figure 3:**
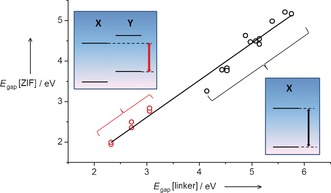
Correlation between the HOCO–LUCO gaps of the ZIF solids (ZnX_2_ or ZnXY) and the HOMO–LUMO gaps of the protonated linkers in gas phase. Red circles correspond to inter‐linker transitions in mixed ZIFs (there are two points for each mixed composition, corresponding to the two ordered configurations). Black circles refer to single‐linker ZIFs.

Finally, it is important to discuss how the electronic properties of the proposed ZIFs are modified by the presence of transition‐metal cations (instead of Zn) in the tetrahedral sites. The established picture of photocatalysis by MOFs requires that photoexcitations involve ligand‐to‐metal charge transfer (LMCT),^18^ because this allows 1) strong light absorption due to the redox nature of the metal center, and 2) effective electron–hole separation to minimize recombination. While the mixed‐linker Zn‐based ZIFs proposed above exhibit a fundamental gap involving charge separation (from one kind of linker to the other), such excitation might not occur from optical absorption, because it would involve charge transfer through the Zn^II^ center, which does not have any empty levels at the same energy range as the LUCO. Furthermore, even if the inter‐linker charge transfer in the Zn‐based mixed ZIFs was possible, the absence of a metal redox center might lead to light absorption that is too weak for the system to work as an efficient photocatalyst.

We have therefore considered the cases of Co or Cu on the tetrahedral sites of the mIm/nIm mixed ZIF. These two metals are chosen because they are known from experiment to incorporate in ZIF structures, and in fact they constitute the metal centers in two reported ZIF photocatalysts.^7^ Figure 2 d shows that the transition metals introduce empty d‐levels above the LUCO level of the ZIF. In the case of Co, the empty d‐band edge is too high, but in the case of Cu the empty d‐band edge is only about 0.6 eV above the LUCO. In fact, in the Cu case the LUCO and the Cu 3d band clearly interact leading to some lowering of the LUCO energy. The presence of Cu and the overlap of its empty 3d levels with the LUCO of the ZIFs have important implications for photocatalysis. The photoadsorption leading to electron excitation would be stronger thanks to the Cu^II^/Cu^I^ redox pair. In addition, the hybrid nature of the conduction edge leads to a delocalization of the excited electron which would compete with recombination to the HOCO, thus increasing the lifetime of the excitation. This picture is consistent with very recent experimental measurements in ZIFs with composition Co(mIm)_2_, where it was observed that the hybrid nature of the excited state led to longer charge carrier lifetimes.^19^ The potential advantage of our mixed‐linker ZIFs is that they offer unique flexibility to simultaneously tune the band gap and achieve overlap between the ZIF LUCO and the empty metal levels.

In summary, this work illustrates a conceptually simple route to engineer the band structure of ZIFs, based on the mixing of linkers within the framework, to target photocatalytic applications. In particular, we have shown that in this way it is possible to design new ZIF materials which 1) are thermodynamically stable with respect to the pure ones, 2) have narrower band gaps, of the required magnitude for solar light absorption, and 3) have band edges in ideal positions for visible‐light water splitting and CO_2_ reduction photocatalysis. We have also shown that via the incorporation of a transition metal (Cu) in the tetrahedral position of the mixed‐linker ZIFs, it might be possible to increase photoadsorption and simultaneously extend the electron–hole recombination times, thanks to the overlap between the empty metal d‐levels and the lowest unoccupied crystal orbitals in the structure. Further theoretical and experimental work is required to establish whether these excitations are indeed optically accessible, as there can be thermodynamic and/or kinetic limitations to the charge transfer process involved in such transitions.

## Supporting information

As a service to our authors and readers, this journal provides supporting information supplied by the authors. Such materials are peer reviewed and may be re‐organized for online delivery, but are not copy‐edited or typeset. Technical support issues arising from supporting information (other than missing files) should be addressed to the authors.

Supplementary

## References

[1] H. Furukawa , K. E. Cordova , M. O'Keeffe , O. M. Yaghi , Science 2013, 341, 1230444. 23990564 10.1126/science.1230444

[2a] J.-L. Wang , C. Wang , W. Lin , ACS Catal. 2012, 2, 2630–2640;

[2b] Y. Li , H. Xu , S. Ouyang , J. Ye , Phys. Chem. Chem. Phys. 2016, 18, 7563–7572; 26535907 10.1039/c5cp05885f

[2c] M. A. Nasalevich , M. van der Veen , F. Kapteijn , J. Gascon , CrystEngComm 2014, 16, 4919–4926.

[3a] C. Wang , Z. Xie , K. E. deKrafft , W. Lin , J. Am. Chem. Soc. 2011, 133, 13445–13454; 21780787 10.1021/ja203564w

[3b] S. Pullen , H. Fei , A. Orthaber , S. M. Cohen , S. Ott , J. Am. Chem. Soc. 2013, 135, 16997–17003; 24116734 10.1021/ja407176p PMC3829681

[3c] C. Wang , K. E. deKrafft , W. Lin , J. Am. Chem. Soc. 2012, 134, 7211–7214; 22486151 10.1021/ja300539p

[3d] J. He , Z. Yan , J. Wang , J. Xie , L. Jiang , Y. Shi , F. Yuan , F. Yu , Y. Sun , Chem. Commun. 2013, 49, 6761–6763; 10.1039/c3cc43218a 23778364

[3e] A. Fateeva , P. A. Chater , C. P. Ireland , A. A. Tahir , Y. Z. Khimyak , P. V. Wiper , J. R. Darwent , M. J. Rosseinsky , Angew. Chem. Int. Ed. 2012, 51, 7440–7444; 10.1002/anie.201202471 22696508

[3f] M. A. Nasalevich , R. Becker , E. V. Ramos-Fernandez , S. Castellanos , S. L. Veber , M. V. Fedin , F. Kapteijn , J. N. H. Reek , J. I. van der Vlugt , J. Gascon , Energy Environ. Sci. 2015, 8, 364–375.

[4] J. Gascon , M. D. Hernandez-Alonso , A. R. Almeida , G. P. M. van Klink , F. Kapteijn , G. Mul , ChemSusChem 2008, 1, 981–983. 19053135 10.1002/cssc.200800203

[5] C. G. Silva , I. Luz , F. X. L. i. Xamena , A. Corma , H. García , Chem. Eur. J. 2010, 16, 11133–11138. 20687143 10.1002/chem.200903526

[6a] Y.-Q. Tian , Y.-M. Zhao , Z.-X. Chen , G.-N. Zhang , L.-H. Weng , D.-Y. Zhao , Chem. Eur. J. 2007, 13, 4146–4154; 17397024 10.1002/chem.200700181

[6b] A. Phan , C. J. Doonan , F. J. Uribe-Romo , C. B. Knobler , M. O'Keeffe , O. M. Yaghi , Acc. Chem. Res. 2010, 43, 58–67; 19877580 10.1021/ar900116g

[6c] B. Chen , Z. Yang , Y. Zhu , Y. Xia , J. Mater. Chem. A 2014, 2, 16811–16831.

[7a] S. Wang , W. Yao , J. Lin , Z. Ding , X. Wang , Angew. Chem. Int. Ed. 2014, 53, 1034–1038; 10.1002/anie.201309426 24339134

[7b] H. Yang , X.-W. He , F. Wang , Y. Kang , J. Zhang , J. Mater. Chem. 2012, 22, 21849–21851.

[8] W. J. Greenlee , in US Patent 5,164,407, 1992.

[9a] J. Heyd , G. E. Scuseria , M. Ernzerhof , J. Chem. Phys. 2003, 118, 8207–8215;

[9b] J. Heyd , G. E. Scuseria , M. Ernzerhof , J. Chem. Phys. 2006, 124, 219906.

[10] K. T. Butler , C. H. Hendon , A. Walsh , J. Am. Chem. Soc. 2014, 136, 2703–2706. 24447027 10.1021/ja4110073 PMC3946036

[11a] K. Hendrickx , D. E. P. Vanpoucke , K. Leus , K. Lejaeghere , A. Van Yperen-De Deyne , V. Van Speybroeck , P. Van Der Voort , K. Hemelsoet , Inorg. Chem. 2015, 54, 10701–10710; 26540517 10.1021/acs.inorgchem.5b01593

[11b] C. H. Hendon , D. Tiana , M. Fontecave , C. Sanchez , L. D′arras , C. Sassoye , L. Rozes , C. Mellot-Draznieks , A. Walsh , J. Am. Chem. Soc. 2013, 135, 10942–10945; 23841821 10.1021/ja405350u

[11c] H. Q. Pham , T. Mai , N.-N. Pham-Tran , Y. Kawazoe , H. Mizuseki , D. Nguyen-Manh , J. Phys. Chem. C 2014, 118, 4567–4577.

[12a] H. Wang , L. Zhang , Z. Chen , J. Hu , S. Li , Z. Wang , J. Liu , X. Wang , Chem. Soc. Rev. 2014, 43, 5234–5244; 24841176 10.1039/c4cs00126e

[12b] M. G. Walter , E. L. Warren , J. R. McKone , S. W. Boettcher , Q. Mi , E. A. Santori , N. S. Lewis , Chem. Rev. 2010, 110, 6446–6473; 21062097 10.1021/cr1002326

[12c] T. Hisatomi , J. Kubota , K. Domen , Chem. Soc. Rev. 2014, 43, 7520–7535. 24413305 10.1039/c3cs60378d

[13] S. Trasatti , Pure Appl. Chem. 1986, 58, 955–966.

[14a] D. K. Kanan , E. A. Carter , J. Phys. Chem. C 2012, 116, 9876–9887;

[14b] S. Hamad , N. C. Hernandez , A. Aziz , A. R. Ruiz-Salvador , S. Calero , R. Grau-Crespo , J. Mater. Chem. A 2015, 3, 23458–23465.

[15] M. A. Nasalevich , C. H. Hendon , J. G. Santaclara , K. Svane , B. van der Linden , S. L. Veber , M. V. Fedin , A. J. Houtepen , M. A. van der Veen , F. Kapteijn , A. Walsh , J. Gascon , Sci. Rep. 2016, 6, 23676. 27020767 10.1038/srep23676 PMC4810359

[16] R. Banerjee , A. Phan , B. Wang , C. Knobler , H. Furukawa , M. O'Keeffe , O. M. Yaghi , Science 2008, 319, 939–943. 18276887 10.1126/science.1152516

[17] R. Banerjee , H. Furukawa , D. Britt , C. Knobler , M. O'Keeffe , O. M. Yaghi , J. Am. Chem. Soc. 2009, 131, 3875–3877. 19292488 10.1021/ja809459e

[18a] Y. H. Fu , D. R. Sun , Y. J. Chen , R. K. Huang , Z. X. Ding , X. Z. Fu , Z. H. Li , Angew. Chem. Int. Ed. 2012, 51, 3364–3367; 10.1002/anie.201108357 22359408

[18b] D. R. Sun , Y. H. Fu , W. J. Liu , L. Ye , D. K. Wang , L. Yang , X. Z. Fu , Z. H. Li , Chem. Eur. J. 2013, 19, 14279–14285. 24038375 10.1002/chem.201301728

[19] B. Pattengale , S. Yang , J. Ludwig , Z. Huang , X. Zhang , J. Huang , J. Am. Chem. Soc. 2016, 138, 8072–8075. 27322216 10.1021/jacs.6b04615

