# Supplementary material for: Modelling a Linker Mix‐and‐Match Approach for Controlling the Optical Excitation Gaps and Band Alignment of Zeolitic Imidazolate Frameworks
Source: Angew Chem Int Ed Engl. 2016 Nov 15;55(52):16012–6. doi: 10.1002/anie.201609439 (PMC5216902; doi:10.1002/anie.201609439)
Supplement: Supplementary file 1 — Supplementary [file ANIE-55-16012-s001.pdf]

Angewandte Chemie
Eine Zeitschrift der Gesellschaft Deutscher Chemiker

Supporting Information

Modelling a Linker Mix-and-Match Approach for Controlling the Optical Excitation Gaps and Band Alignment of Zeolitic Imidazolate Frameworks

Ricardo Grau-Crespo, Alex Aziz, Angus W. Collins, Rachel Crespo-Otero, Norge C. Hernández, L. Marleny Rodriguez-Albelo, A. Rabdel Ruiz-Salvador, Sofia Calero, and Said Hamad**

anie_201609439_sm_miscellaneous_information.pdf

Supporting Information

1. Methodology

The ZIF solids were represented by primitive rhombohedral cells, containing 6 ZnX_2 formula units, where X stands for the imidazolate-based linker. The DFT calculations of the periodic structures were performed using VASP.[1] A cutoff of 520 eV was used for the plane-wave expansion. Considering the large real-space dimensions of the cells, and the expected weak band dispersion, only the Γ point of the Brillouin zone was used in the calculations. In a first step, full geometry optimizations were performed, including both atom positions and lattice parameters. No symmetry constraints were applied, and we started from rhombohedral cells with the cell angle slightly distorted from the special value (109.47°) corresponding to the body-centered cubic (bcc) structure. The reason for this is that, while some ZIFs with SOD topology retain the ideal cubic structure (e.g. $Zn(mIm)_2$), others are known from experiment to distort into a less symmetric hexagonal form (e.g. $Zn(bIm)_2$), which corresponds to an angle different from 109.47° in the primitive rhombohedral cell.[2] Geometry optimizations were performed with the PBE functional,[3] corrected with Grimme's D2 method for including dispersion interactions[4] (test calculations showed that the use of more sophisticated dispersion corrections brought only negligible change both on the geometry and the final electronic structure). In a second step, accurate electronic structures were obtained from single-point calculations with the HSE06 screened hybrid functional,[5,6] which generally provides band-gaps in close agreement with experiment,[7] and has been shown to perform well in electronic structure calculations of MOFs.[8,9]

In the case of Co-containing ZIFs, both low-spin and high-spin configurations were tried, in order to identify the groundstate (high-spin). Electron energy levels calculated with periodic DFT codes like VASP are obtained with respect to an internal reference (typically the average potential in the crystal). In order to align the electronic energies with the vacuum scale, it is necessary to calculate the electrostatic potential in a pseudo-vacuum region within the simulation cell. We follow here the methodology recently proposed by Butler et al.[8] to calculate the vacuum level in MOF structures, by evaluating the average potential within a small sphere (radius of 2 Å) at the pore (see Supporting Information). In Ref. [8], this procedure led to ZIF-8 ionization potentials in good agreement with experiment. A Python code provided by these authors was employed in our calculations to obtain the average potentials. The electronic structures of the linkers in molecular form (with an attached proton, i.e. HX) were calculated using Gaussian09.[10] As in the case of the ZIF bulk calculations, the electronic structures of the molecules were obtained from single-point calculations using the HSE06 functional at PBE-optimized geometries. Wavefunctions were expanded using the aug-cc-pVTZ basis set.

2. Cell parameters of optimized crystal structures

Table S1 shows the optimized cell parameters of the cells, given in the hexagonal setting for convenience. Note that some of the structures (in bold font in the Table) exhibit a small distortion from the hexagonal symmetry.

Table S1: Optimized lattice parameters for the hexagonal cells of ZIF crystal structures with SOD topology.

linker	*a* (Å)	*b* (Å)	*c* (Å)	*α* (°)	*β* (°)	*γ* (°)
bIm	22.37	22.37	16.09	90.00	90.00	120.00
dcIm	22.44	22.44	16.04	90.00	90.00	120.00
dcnIm	23.16	23.16	16.12	90.00	90.00	120.00
fIm	21.81	21.81	15.90	90.00	90.00	120.00

Ica	24.29	24.29	14.91	90.00	90.00	120.00
Im	22.38	22.38	16.23	90.00	90.00	120.00
mIm	22.83	22.83	15.94	90.00	90.00	120.00
nIm	24.30	24.30	14.51	90.00	90.00	120.00
tIm	22.52	22.52	15.87	90.00	90.00	120.00
**abIm**	**22.28**	**22.42**	**16.05**	**90.28**	**90.06**	**120.03**
**acIm**	**22.09**	**22.14**	**15.96**	**90.32**	**89.92**	**120.11**
**pur**	**22.03**	**22.17**	**16.02**	**90.25**	**90.10**	**120.29**
fIm/nIm	24.63	24.63	13.64	90.00	90.00	120.00
Im/nIm	22.72	22.72	16.24	90.00	90.00	120.00
mIm/nIm	24.96	24.96	14.30	90.00	90.00	120.00

3. Relative energies of the mixed configurations

We explain here how we generated the distribution of linkers in the mixed configurations. Since there are 12 linker sites in the primitive rhombohedral cell, the mixed solids with composition ZnXY can arrange the 6 X and 6 Y linkers in many possible configurations. We have considered two well-ordered structures for each composition, in such a way that the symmetry of the mixed configurations is maximized, and then picked up the more stable of the two for further analysis. The two ordered configurations are defined in terms of the symmetrically distinct linker sites in a structure with SOD topology and hexagonal distortion, as explained below. Choosing the ordered configurations is justified not only because it speeds up calculations: there is experimental evidence that mixed-linker ZIFs exhibit an ordered distribution of linkers, in such a way that each linker occupies a distinct crystallographic site.[11] Since in our work we have considered the most stable ordered configuration of this kind for each mixed-linker ZIF, it is likely that our calculated configurations will correspond to the ones that would be obtained in experiment. In any case, as our work shows, the bandgaps and band edge positions of this family of ZIFs depend mainly on which linkers are present, and therefore the distribution of linkers in the mixed framework during the simulation should not have any significant effect on our conclusions.

We now explain the symmetrically distinct linker sites in a structure with SOD topology. In the “undistorted” cubic SOD structure (Figure S1a), all linker sites are equivalent. In the absence of any additional symmetry breaking (e.g. due to the molecular structure of the linker), the structure has 4 three-fold rotation axes along the cube diagonals, which define the main pores of the SOD topology (Figure S1b). The primitive cell corresponding to the cubic SOD structure is a rhombohedrum with cell angle 109.47°. The hexagonal distortion occurs when the rhombohedral angle deviates from this ideal value, which leads to the hexagonal conventional cell in Figures S1c,d. Now, there are two symmetrically distinct linker sites, represented by blue and red spheres in the figure. Only one of the 4 main pore directions conserves the three-fold rotation symmetry, which is the one perpendicular to the basal plane of the hexagonal structure (Figure S1c). The rings defining this unique pore contain six symmetrically equivalent anion sites (blue in the figure). In contrast, the other three pores contain a mixture of 4 red and 2 blue sites in the ring, and do not have the original 3-fold rotation symmetry. It is clear then that the maximally ordered structures with 50:50 mixture of anions are the ones where each anion species occupies only one symmetrically distinct site. There are two inequivalent configurations of this type, which do not break the symmetry of the hexagonal lattice. Since we always use nIm as one of the linkers in the mixed structures, we call **configuration 1** to the configuration where only the nIm linker is in the unique-pore rings, and **configuration 2** to the inverted configuration.

Table S2 shows the mixing energies, calculated as:

$$E_{\text{mix}}[\text{ZnXY}] = E[\text{ZnXY}] - \frac{1}{2}E[\text{ZnX}] - \frac{1}{2}E[\text{ZnY}]$$

Table S2: Mixing energies of the two maximally-ordered configurations of mixed ZIFs, as obtained from HSE calculations (in meV per formula unit). Underlined values mark the most stable configuration in each case.

Mixed ZIF	E_{mix}[Configuration 1]	E_{mix}[Configuration 1]
Zn(nIm)(Im)	-238	-362
Zn(nIm)(mIm)	+41	-358
Zn(nIm)(fIm)	-228	+315

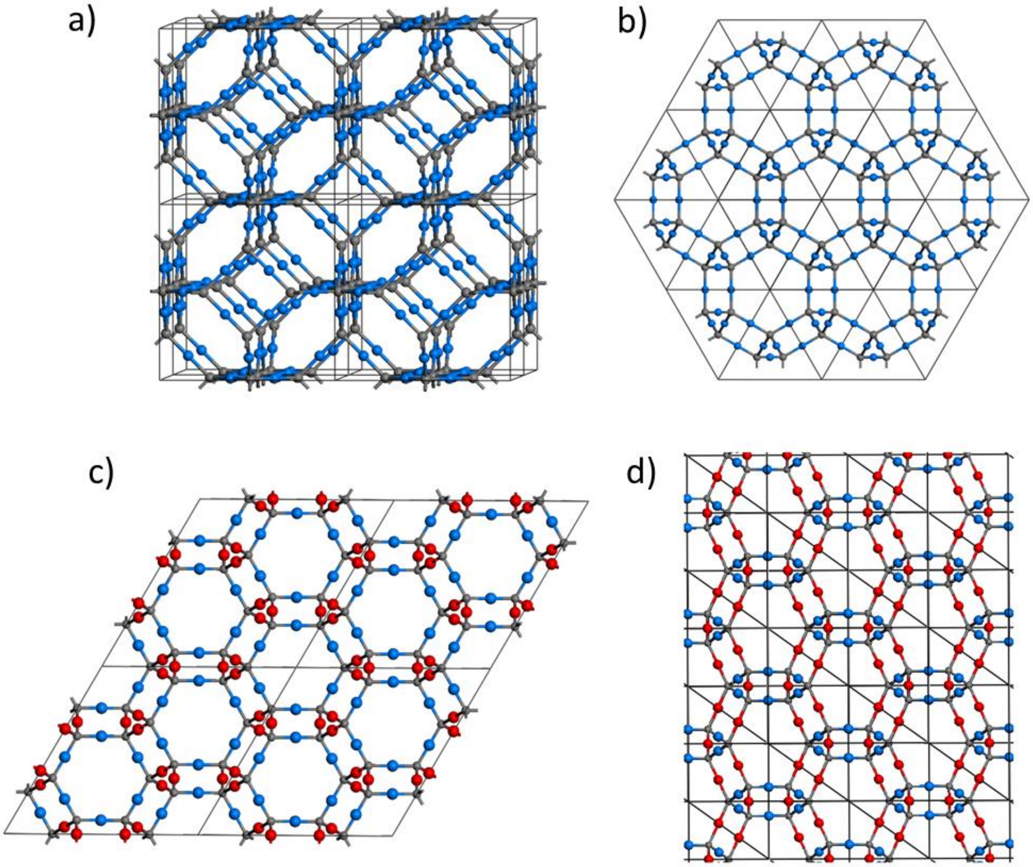

Figure S1. Schematic representation of the SOD topology with cubic structure (a, b) and with hexagonal structure (c, d). Gray spheres represent the cations (Zn in our case) and blue or red represent the anion (imidazolate linkers in our case). Two anion colors are used to indicate the symmetrically distinct anion sites in the hexagonal structure.

4. Projected density of states

Figure S2 shows the electronic density of states (DOS) for the two mixed Zn-based ZIFs with compositions Zn(mIm)(nIm) and Zn(fIm)(nIm). The energy scales are aligned using the vacuum levels as reference. The DOS illustrates the origin of the band edges in the mixed ZIFs.

It is clear that in the mixed Zn(mIm)(nIm) solid, the conduction band edge is mainly contributed by the nitro groups in the nIm linkers (as in the pure $Zn(nIm)_2$), while the valence band edge is contributed by the ring carbon atoms in the mIm linker (as in the pure $Zn(mIm)_2$). Analogously, in the mixed Zn(fIm)(nIm) solid, the conduction band edge is again contributed by the nitro groups in the nIm linkers, while the valence band edge is contributed by the fIm linker (as in the pure $Zn(fIm)_2$).

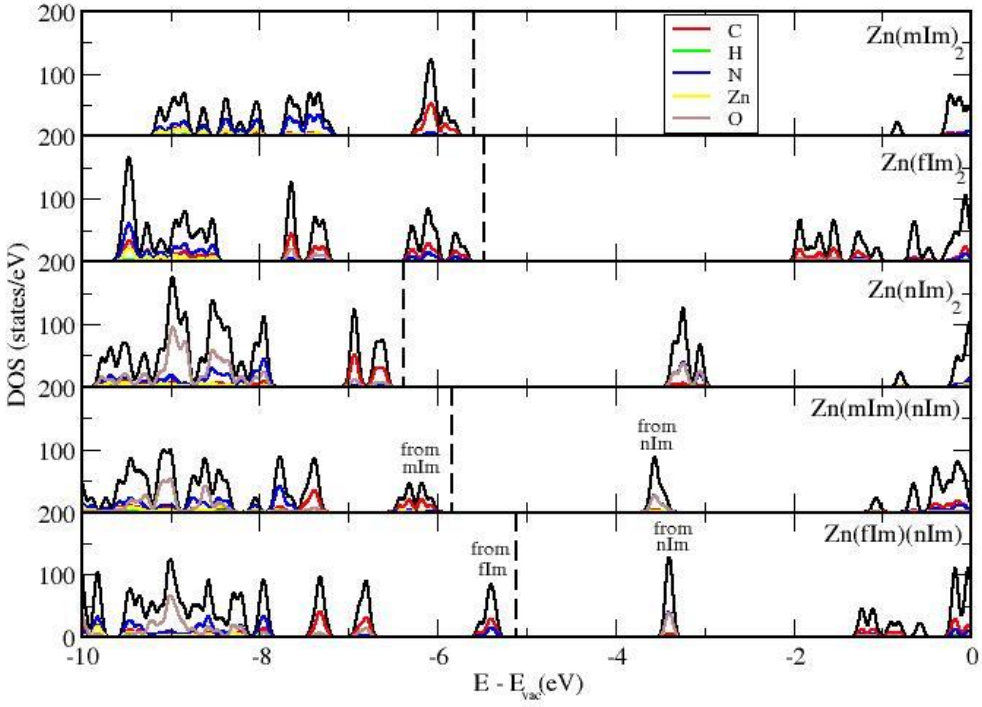

Figure S2. Total and projected electronic density of states (DOS) corresponding to single-linker ZIF structures $Zn(mIm)_2$, $Zn(nIm)_2$, $Zn(fIm)_2$ and to the mixed-linker ZIF structures Zn(mIm)(nIm) and Zn(mIm)(fIm). The vertical dashed lines represent the Fermi levels in each case.

Figure S3 shows the projected DOS for the Cu- and Co-containing mixed ZIFs, indicating the positions of the transition-metal 3d levels. These are spin-polarized electronic structures which have been calculated here using a simple ferromagnetic ordering of the spin moments. In reality, these structures are likely to be paramagnetic as one would expect very weak magnetic coupling through the imidazolate linkers. The most stable spin configurations of the Co(II) metal centers was found to be high-spin (S=3/2).

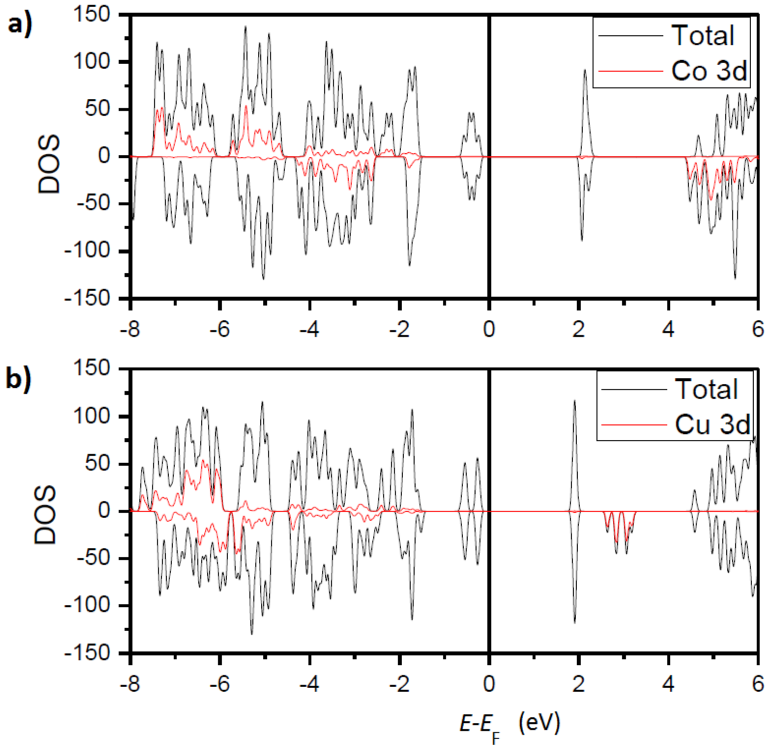

Figure S3. Total and 3*d*-projected electronic density of states (DOS) corresponding mixed-linker ZIF structures a) Co(mIm)(nIm) and b) Cu(mIm)(nIm).

5. Estimation of the vacuum energy level

Following the procedure proposed by Butler et al.[8] we use the potential at the center of the largest pore (the one that conserves the 3-fold rotational symmetry after hexagonal distortion) as an approximation to the vacuum level. The potential is averaged in a sphere of radius 2 Å. The resulting average is plotted, in Figure S4, as a function of the coordinate of the center of the sphere along the pore, i.e. the value of x in the coordinate (x,x,x) within the rhombohedral cell. The electrostatic potential at (0,0,0) is taken as an approximation to the vacuum level. In all cases this is a point of zero gradient (no electric field), and we have checked that in most of the structures it is also the point farthest apart from the framework atoms.

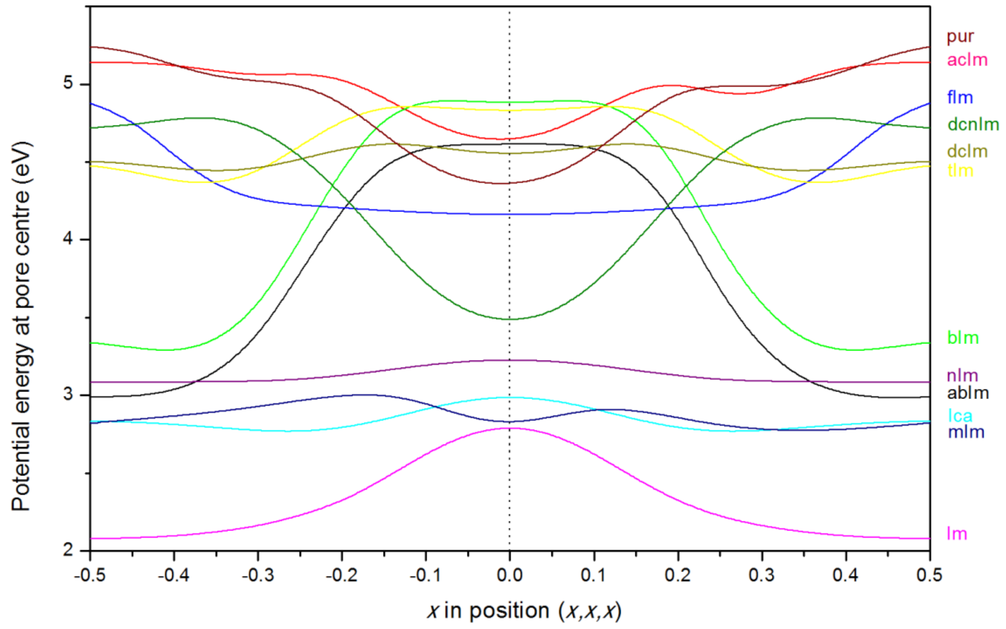

Figure S4: Electrostatic potential variation across the direction (111) at the pore center.

6. HOMO-LUMO positions and gaps for imidazole-based linkers

Finally, we provide in Table S3 a list of 42 modified imidazolate linkers, and their calculated their HOMO and LUMO, which can serve as a starting point for future design of mixed-linker ZIFs with tailored bands. Only the most stable configuration is showed whenever there are different ones for a given composition. These calculations were performed with Gaussian-09.[4]

Table S3: List of calculated HOMO-LUMO of modified imidazole linkers, sorted in decreasing order of band gap.

Linker	Molecular structure	HOMO (eV)	LUMO (eV)	Gap (eV)
cnIm	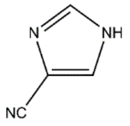	-7.06	-1.25	5.81
Im	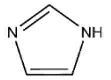	-6.32	-0.57	5.76
dcIm	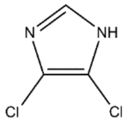	-6.37	-0.74	5.63
cIm	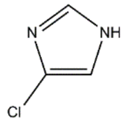	-6.35	-0.73	5.62
cneIm	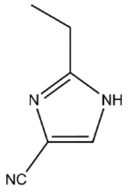	-6.70	-1.10	5.60
brIm	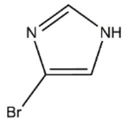	-6.31	-0.78	5.53
mIm	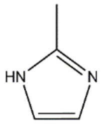	-5.99	-0.57	5.42
eIm	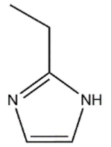	-5.96	-0.56	5.41
cmIm	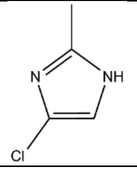	-6.06	-0.72	5.34
ceIm	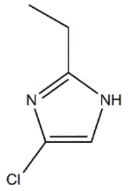	-6.05	-0.73	5.32

Linker	Molecular structure	HOMO (eV)	LUMO (eV)	Gap (eV)
dbrIm	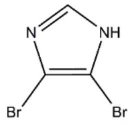	-6.33	-1.01	5.32
brmIm	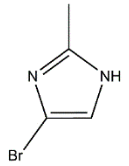	-6.04	-0.74	5.30
breIm	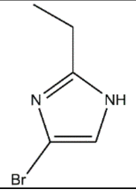	-6.02	-0.75	5.28
cnmIm	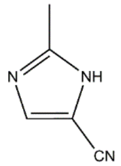	-6.76	-1.54	5.22
acIm	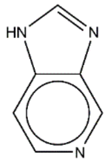	-6.57	-1.40	5.17
bIm	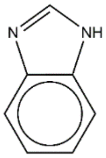	-6.24	-1.10	5.15
pur	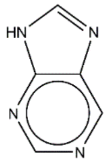	-7.07	-1.93	5.13
mbIm	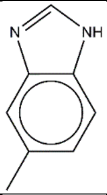	-6.08	-1.02	5.05
dmbim	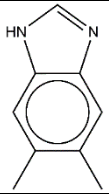	-5.90	-0.93	4.98

Linker	Molecular structure	HOMO (eV)	LUMO (eV)	Gap (eV)
abIm	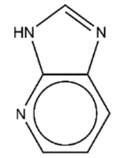	-6.59	-1.62	4.97
dcnIm	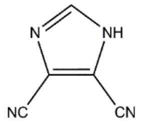	-7.67	-2.79	4.88
cbIm	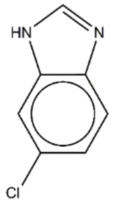	-6.27	-1.41	4.87
brbIm	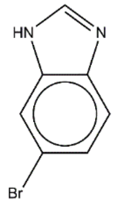	-6.26	-1.44	4.82
dcbIm	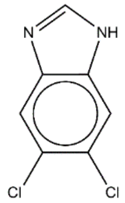	-6.44	-1.66	4.78
pIm	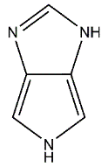	-5.17	-0.43	4.74
dbrbIm	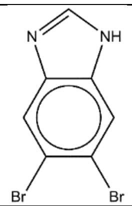	-6.42	-1.69	4.73
fIm	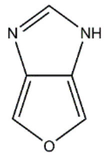	-5.59	-1.06	4.53
Ica	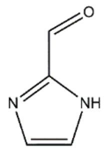	-6.90	-2.42	4.49

Linker	Molecular structure	HOMO (eV)	LUMO (eV)	Gap (eV)
cnIca	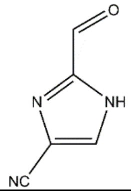	-7.51	-3.06	4.46
tIm	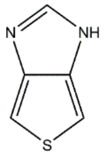	-5.78	-1.35	4.43
cIca	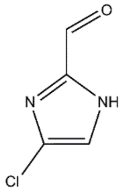	-6.87	-2.70	4.17
nIm	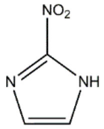	-7.41	-3.27	4.14
cnnIm	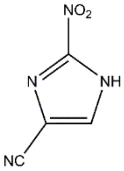	-7.94	-3.84	4.10
brIca	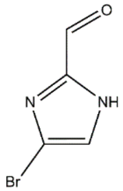	-6.81	-2.71	4.10
nbIm	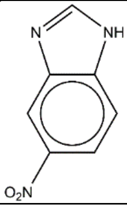	-7.05	-2.99	4.06
dcnnIm	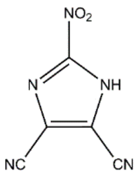	-8.41	-4.42	4.00
clnIm	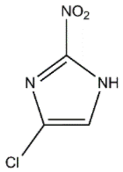	-7.29	-3.50	3.79

Linker	Molecular structure	HOMO (eV)	LUMO (eV)	Gap (eV)
brnIm	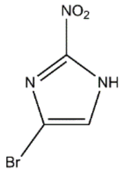	-7.19	-3.50	3.69
dclnIm	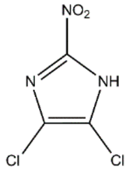	-7.24	-3.62	3.61
dbrnIm	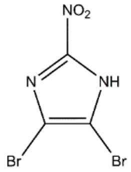	-7.13	-3.61	3.52
clnIm	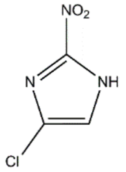	-7.29	-3.50	3.79
brnIm	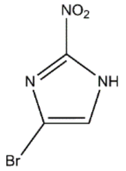	-7.19	-3.50	3.69

References

(1) Kresse, G.; Furthmüller, J. *Phys. Rev. B* **1996**, *54*, 11169.

(2) Park, K. S.; Ni, Z.; Côté, A. P.; Choi, J. Y.; Huang, R.; Uribe-Romo, F. J.; Chae, H. K.; O'Keeffe, M.; Yaghi, O. M. *Proc. Nat. Acad. Sci.* **2006**, *103*, 10186.

(3) Perdew, J. P.; Burke, K.; Ernzerhof, M. *Phys. Rev. Lett.* **1996**, *77*, 3865.

(4) Grimme, S. *J. Comput. Chem.* **2006**, *27*, 1787.

(5) Heyd, J.; Scuseria, G. E.; Ernzerhof, M. *J. Chem. Phys.* **2003**, *118*, 8207.

(6) Heyd, J.; Scuseria, G. E.; Ernzerhof, M. *J. Chem. Phys.* **2006**, *124*, 219906.

(7) Henderson, T. M.; Paier, J.; Scuseria, G. E. *Phys. Status Solidi B* **2011**, *248*, 767.

(8) Butler, K. T.; Hendon, C. H.; Walsh, A. *J. Am. Chem. Soc.* **2014**, *136*, 2703.

(9) Ling, S.; Slater, B. *J. Phys. Chem. C* **2015**, *119*, 16667.

(10) Frisch, M. J.; Trucks, G. W.; Schlegel, H. B.; Scuseria, G. E.; Robb, M. A.; Cheeseman, J. R.; Scalmani, G.; Barone, V.; Mennucci, B.; Petersson, G. A.; Nakatsuji, H.; Caricato, M.; Li, X.; Hratchian, H. P.; Izmaylov, A. F.; Bloino, J.; Zheng, G.; Sonnenberg, J. L.; Hada, M.; Ehara, M.; Toyota, K.; Fukuda, R.; Hasegawa, J.; Ishida, M.; Nakajima, T.; Honda, Y.; Kitao, O.; Nakai, H.;

Vreven, T.; Montgomery Jr., J. A.; Peralta, J. E.; Ogliaro, F.; Bearpark, M. J.; Heyd, J.; Brothers, E. N.; Kudin, K. N.; Staroverov, V. N.; Kobayashi, R.; Normand, J.; Raghavachari, K.; Rendell, A. P.; Burant, J. C.; Iyengar, S. S.; Tomasi, J.; Cossi, M.; Rega, N.; Millam, N. J.; Klene, M.; Knox, J. E.; Cross, J. B.; Bakken, V.; Adamo, C.; Jaramillo, J.; Gomperts, R.; Stratmann, R. E.; Yazyev, O.; Austin, A. J.; Cammi, R.; Pomelli, C.; Ochterski, J. W.; Martin, R. L.; Morokuma, K.; Zakrzewski, V. G.; Voth, G. A.; Salvador, P.; Dannenberg, J. J.; Dapprich, S.; Daniels, A. D.; Farkas, Ö.; Foresman, J. B.; Ortiz, J. V.; Cioslowski, J.; Fox, D. J.; Gaussian, Inc.: Wallingford, CT, USA, 2009. Gaussian 09.

(11) Banerjee, R.; Furukawa, H.; Britt, D.; Knobler, C.; O'Keeffe, M.; Yaghi, O. M. *J. Am. Chem. Soc.* **2009**, *131*, 3875.
